# Unmanned-Aerial-Vehicle Trajectory Planning for Reliable Edge Data Collection in Complex Environments

**DOI:** 10.3390/biomimetics10020109

**Published:** 2025-02-12

**Authors:** Zhengzhe Xiang, Fuli Ying, Xizi Xue, Xiaorui Peng, Yufei Zhang

**Affiliations:** 1 Shcool of Computer and Computing Science, Hangzhou City University, Hangzhou 310025, China; xiangzz@hzcu.edu.cn (Z.X.); yingfuli@stu.hzcu.edu.cn (F.Y.); 32201055@stu.hzcu.edu.cn (X.P.); 2 College of Computer Science and Technology, Zhejiang University, Hangzhou 310007, China; xuexizi@zju.edu.cn; 3 Shcool of Art and Archeology, Hangzhou City University, Hangzhou 310025, China

**Keywords:** trajectory optimization, data collection, edge computing, low-altitude economy

## Abstract

With the rapid advancement of edge-computing technology, more computing tasks are moving from traditional cloud platforms to edge nodes. This shift imposes challenges on efficiently handling the substantial data generated at the edge, especially in extreme scenarios, where conventional data collection methods face limitations. UAVs have emerged as a promising solution for overcoming these challenges by facilitating data collection and transmission in various environments. However, existing UAV trajectory optimization algorithms often overlook the critical factor of the battery capacity, leading to potential mission failures or safety risks. In this paper, we propose a trajectory planning approach Hyperion that incorporates charging considerations and employs a greedy strategy for decision-making to optimize the trajectory length and energy consumption. By ensuring the UAV’s ability to return to the charging station after data collection, our method enhances task reliability and UAV adaptability in complex environments.

## 1. Introduction

Edge computing is a distributed computing paradigm that processes data closer to its source, such as IoT devices or local edge servers [1]. In this paradigm, computational tasks are prioritized to be completed at the edge. With the vigorous development and maturation of edge computing technology, an increasing number of tasks are being shifted from traditional cloud-computing platforms to edge nodes [2]. For instance, in autonomous driving vehicles, real-time processing of sensor data is crucial for making driving decisions [3]. These sensors include radar, LiDAR, cameras, etc. [4], generating large amounts of data with high real-time requirements. By migrating computing tasks to edge-computing units within vehicles, the latency of transmitting data to the cloud can be significantly reduced, thereby enhancing the vehicle’s responsiveness and safety in complex traffic environments [5].

There are many examples of optimizations specifically aimed at computational tasks of terminal devices. Some research has proposed a standardized optimization method to port DNNs to embedded devices, in order to enhance the computational speed of terminal devices [6]. Graziosi et al. have deployed a neural network based on smart cameras to detect crowd congestion conditions in real time [7]. However, this shift in the computing location also results in a substantial amount of data being generated at the edge. These data generated at the edge often need to be promptly collected and processed to meet various application requirements. Typically, edge nodes upload their data to edge servers they are connected to, and these dispersed data are aggregated through the hierarchy of end devices–edge servers–cloud-computing platforms [8]. However, not all application scenarios allow data to be collected smoothly in this manner. In many extreme scenarios, the network quality is poor or there are no edge servers assisting end devices in proximity [9] (such as in remote areas and at natural disaster sites), the wireless network coverage is limited, or a reliable connection cannot be established, posing significant challenges to data transmission. Therefore, efficiently recovering data generated by edge nodes in extreme scenarios has become an urgent problem in need of a solution.

Recently, UAVs have been widely used as a flexible and efficient means of transportation in high-altitude filming or short-distance rapid-delivery scenarios, being considered as a core element in the development of the “low-altitude economy” [10]. Their high maneuverability allows the performance of data collection and retrieval tasks in various complex environments. The data-processing capabilities provided by the computing and storage units they carry have led to UAVs being regarded as a mobile data-processing platform in many research works, attracting extensive attention from researchers [11]. Therefore, in the context of extreme edge data collection challenges, utilizing UAVs to overcome the limitations of traditional wireless network transmission can be considered. For instance, UAVs can use wireless transmission methods, such as Bluetooth and LoRa, to achieve data transmission between terminal nodes and themselves. By planning reasonable UAV flight paths and enabling periodic interactions between UAVs and edge nodes, data can be ensured to be transferred to processing centers in a timely and secure manner.

However, existing UAV trajectory optimization algorithms for data collection typically focus on obtaining the shortest distance or minimum time [12,13,14], neglecting the objective condition that the battery capacity may not meet the energy consumption requirements for one-time data collection in practical scenarios. This oversight may lead to situations where the UAV runs out of battery power on the return trip, rendering it as unable to complete the task or even causing the UAV to crash, resulting in equipment damage and safety hazards. Therefore, in the trajectory-planning process, it is essential to consider the UAV’s battery level as a necessary condition for trajectory decision-making to enhance the UAV’s usability and safety.

In this paper, we will consider recharging in trajectory planning and utilize a greedy strategy [15] for trajectory-point decision-making to effectively optimize the length and energy consumption of the trajectory. This not only ensures that the UAV has sufficient battery power to return to the charging station after completing the data collection task, thus, effectively avoiding the issue of running out of battery power, but also enhances the reliability of the task execution and strengthens the adaptability of UAVs in complex environments.

The methodology proposed in this paper aligns with the principles of biomimetics, drawing inspiration from biological systems to address the challenges of UAV trajectory planning in resource-constrained environments. Similarly, as biological organisms adapt to environmental constraints through efficient resource allocation and optimized movement strategies, the UAV trajectory optimization approach presented in this study emphasizes adaptive energy management and path planning in resource-constrained environments. By mimicking the natural principles of energy efficiency and adaptability, the proposed framework enhances UAV performance in extreme edge data collection scenarios. This biologically inspired perspective not only improves the reliability and safety of UAV operations but also contributes to the broader field of bio-inspired robotics by offering innovative solutions that integrate energy optimization and adaptive path planning.

## 2. Related Works

### 2.1. Trajectory Planning for Energy-Saving Data Collection

Energy efficiency in UAV trajectory planning is a vital research area, especially for data collection in wireless sensor networks (WSNs) and massive machine-type communications (mMTC). Li et al. [16] propose a two-level deep-reinforcement-learning framework for online trajectory planning in dynamic environments, focusing on reducing energy consumption through lightweight network designs suitable for low-power UAVs. Similarly, Zhu et al. [17] introduce a hybrid hovering position selection (HHPS) algorithm and a cuckoo-search-based trajectory planning method, optimizing energy consumption and throughput in mMTC networks.

In efforts to minimize energy consumption, Zhu et al. [18] employ a deep-reinforcement-learning technique called pointer network-A*, which efficiently learns UAV trajectory policies to minimize energy use in UAV-aided WSNs. Additionally, Liu et al. [19] tackle the Age-of-Information (AoI) minimization problem, proposing dynamic programming and genetic algorithm approaches to achieve age-optimized trajectories, indirectly impacting energy consumption by optimizing flight paths.

Existing algorithms typically overlook battery capacity constraints as a hard requirement. Battery capacity is a critical factor for the endurance of UAVs in practical applications. Overlooking this limitation can result in operational failures because of insufficient power during mission execution. Our algorithm incorporates battery capacity limits as a hard constraint, ensuring mission completion while maximizing the operational duration of the UAV.

### 2.2. Trajectory Planning for Fast Data Collection

For time-sensitive applications, fast data collection using UAVs is crucial. Samir et al. [12] address this by optimizing UAV trajectories and radio resource allocation, aiming to maximize the number of served IoT devices within their target upload deadlines. They employ a branch-reduce-and-bound (BRB) algorithm and propose a sub-optimal approach for larger networks, ensuring timely data collection. To enhance data collection speed, Fan et al. [20] propose a bi-directional APF-RRT* algorithm with goal-biased strategies, improving convergence rates and search efficiency and facilitating faster data acquisition. Zhu et al. [21] leverage a transformer-based model combined with a weighted A* algorithm to minimize the total AoI, ensuring the UAV efficiently collects fresh data from IoT networks. In disaster scenarios, Javed et al. [22] and Demiane et al. [23] develop UAV-trajectory-planning approaches focusing on rapid deployment and data collection. These methods ensure effective UAV utilization in emergencies, highlighting the importance of timely UAV operations in critical situations.

These studies collectively underscore advancements in UAV trajectory planning, emphasizing both energy efficiency and speed, tailored to specific operational demands and environmental conditions.

## 3. System Model and Problem Description

Consider a scenario where there are *N* end devices designated for local data processing, awaiting data collection. Each end device, denoted as i∈1,…,N, may have a specific position $P_{i}=\left(x_{i},y_{i}\right)$ located at height $H_{i}$ above it for data collection purposes. When collecting data, a UAV will hover in a circular path around $P_{i}$ at a radius of $r_{i}$ with a speed of $V_{i}$, establishing communication with end device *i* until all the data are gathered. And the UAV achieves the maximum energy efficiency, transmitting the most data within 1 J of energy with the following settings [24]:

(1) $$\begin{array}{cc} r_{i} & =\sqrt{\frac{3c_{2}}{8c_{1}g^{2}}}{\left[{\left(1+\frac{16H_{i}^{2}c_{1}g^{2}}{9c_{2}}\right)}^{1/2}-1\right]}^{1/2},\\ V_{i} & ={\left(\frac{c_{2}m^{2}}{3(c_{1}+c_{2}m^{2}/\left(g^{2}r_{i}^{2}\right))}\right)}^{1/4} \end{array}$$

where *g* is the gravitational acceleration, *m* is the UAV’s mass, and $c_{1}$ and $c_{2}$ are constants related to the environment. Additionally, $c_{1}$ represents the parasitic drag of the UAV, which is a combination of multiple drag components, and it is related to the wing area of the UAV. Furthermore, $c_{2}$ is referred to as lift-induced drag, which is the drag generated because of the wing’s alteration of the airflow. It is related to the UAV’s mass (*m*). To simplify, we introduce $P_{0}$ as the initial landing and charging spot for the UAV. Throughout a data collection cycle, the UAV’s trajectory can be represented by ϕ, initially set as $\phi =(P_{0})$ as the starting point. As the UAV navigates to $P_{i}$ to connect with end device *i* and retrieve the data stored with volume $d_{i}$, the position is recorded to extend the path (ϕ). Herein, the UAV mainly uses long-range wireless transmission methods to collect data from end device *i*.

Following a predetermined strategy (π), denoted as π(i)=j, the UAV will navigate to position $P_{j}=\left(x_{j},y_{j}\right)$ located at a height of $H_{j}$ if it determines that collecting data from end device *j* is optimal in the next step after $P_{i}$. Simultaneously, the new destination ($P_{j}$) will be incorporated into the path (ϕ) as follows:

(2) $$\phi \leftarrow (\phi ,P_{j})$$

This sequence continues until all the end devices have been visited and connected by the UAV, unless the UAV runs out of energy, triggering the “charging” process.

The energy consumption for traveling from position $P_{i}$ to position $P_{j}$ is represented as $c_{i,j}^{F}$, which can be derived from [24]

(3) $$\begin{array}{cc} c_{i,j}^{F} & =\int_{t=0}^{T_{i,j}}[c_{1}\left|\right|v_{i,j}\left(t\right){\left|\right|}^{3}+\frac{c_{2}m^{2}}{\left|\right|v_{i,j}\left(t\right)\left|\right|}\\ + & \frac{c_{2}m^{2}}{\left|\right|v_{i,j}\left(t\right)\left|\right|}\cdot \left(|\right|a_{i,j}{\left(t\right)||}^{2}-\frac{{\left(a_{i,j}\left(t\right)\cdot v_{i,j}\left(t\right)\right)}^{2}}{\left|\right|v_{i,j}\left(t\right){\left|\right|}^{2}})\;] \end{array}$$

where $v_{i,j}\left(t\right)$ and $a_{i,j}\left(t\right)$ denote the velocity and acceleration of the UAV at time *t* during the flight from $P_{i}$ to $P_{j}$. Conversely, upon reaching $P_{j}$, the energy consumption for hovering is calculated using the function

(4) $$c_{j}^{H}=\frac{d_{j}}{W_{j}log\left(1+\frac{\gamma_{j}}{H_{j}^{2}+r_{j}^{2}}\right)}\left[\left(c_{1}+\frac{c_{2}m^{2}}{g^{2}r_{j}^{2}}\right)V_{j}^{3}+\frac{c_{2}m^{2}}{V_{j}}\right]$$

where $W_{j}$ represents the network bandwidth, and $\gamma_{j}$ is the reference’s received signal-to-noise ratio (SNR). The total energy consumption for the UAV’s journey between $P_{i}$ and $P_{j}$ can be expressed as

(5) $$c_{i,j}=c_{i,j}^{F}+c_{j}^{H}$$

It is evident that the value of $c_{i,j}$ will be constant and can be computed offline in advance when $P_{i}$ and $P_{j}$ are fixed. This is because the optimal flight trajectory can always be found to derive the best $a_{i,j}\left(t\right)$ and $v_{i,j}\left(t\right)$ from it. Additionally, when the network bandwidth ($B_{j}$) is considered as fixed, the total energy consumption during hovering remains constant.

Thus, with the energy consumption matrix denoted as $c={\left\{c_{i,j}\right\}}_{i=0,j=0}^{N,N}$, the current UAV battery level ($B_{cur}$) and the accumulated energy usage ($B_{sum}$) are updated according to the equations

(6) $$B_{cur}=B_{cur}-c_{i,j},\;B_{sum}=B_{sum}+c_{i,j}$$

after traversing from location $P_{i}$ to $P_{j}$, where $P_{j}$ is included in the set ϕ.

The problem’s objective is now evident: the formulation of the optimal policy (π) to minimize $B_{sum}$, i.e., to achieve the least energy consumption.

## 4. Problem Analysis and Approach

### 4.1. Hyperion Algorithm

As previously mentioned, because of the energy constraints of the UAV, it cannot return to the initial location ($P_{0}$) if the battery energy is insufficient. Hence, a trigger is implemented to guide the UAV to return for recharging before proceeding to collect data from the subsequent end device. The ‘charging’ command for the UAV becomes activated when

(7) $$B-c_{i,j}-c_{j,0}\leq \kappa B^{★},\;\forall P_{j}\in \tilde{P}$$

where $B^{★}$ signifies the UAV battery’s capacity, and κ∈[0,1] denotes the safety margin. This condition signifies that after collecting data at $P_{i}$, the UAV might not have sufficient battery capacity to return to recharge when traveling to $P_{j}$ next, potentially leading to a crash.

Based on the consideration above, the Hyperion (Algorithm 1) is proposed for the core scheduling of the entire UAV flight mission, tasked with planning the optimal flight path while considering battery constraints. Hyperion comprises the following steps:

**(1) Initialization:** In this stage, it identifies all the edge nodes (i.e., data collection points where the UAV may go) then assesses the UAV’s maximum battery capacity and current energy level. The UAV is positioned at the starting location (typically a charging station or a base station).

**(2) Target node evaluation:** In this stage, Hyperion evaluates the energy required to reach each unvisited edge node from the current position and checks if the remaining energy is sufficient to complete the data collection and return to the starting point (ENavi). If the conditions are met, Hyperion explores all the possible paths accessible from that node until the UAV can no longer progress or the battery runs out of energy.

**(3) Optimal path evaluation:** For each path option provided by Hyperion, the number of nodes the UAV can visit along that path and the remaining energy after completing the task are calculated. Following a greedy strategy, the path that allows the UAV to visit the most nodes with sufficient remaining energy is chosen to achieve more efficient energy utilization and greater data collection.

**(4) Charging determination:** If no feasible path is available (e.g., insufficient energy to reach any other node) or all the nodes have been visited, the UAV returns to the starting point for recharging. After each return, the UAV undergoes a full recharge in preparation for the next flight mission.

**(5) Flight plan update:** After each flight, Hyperion updates the list of visited nodes and records the flight path and total energy consumption. Task plans are adjusted based on actual conditions to avoid revisiting the same nodes.

The above process continues in a loop until all the designated edge nodes have been visited, ensuring that the UAV maximizes data collection within the allowable battery range while always ensuring a safe return.

Within the Hyperion algorithm, we can find that the ENavi algorithm serves as the core algorithm for UAV path planning, aiding UAVs in exploring the longest path from a given node and eventually returning to the starting point. The details of this are shown in Algorithm 2.

**Algorithm 1:** Hyperion

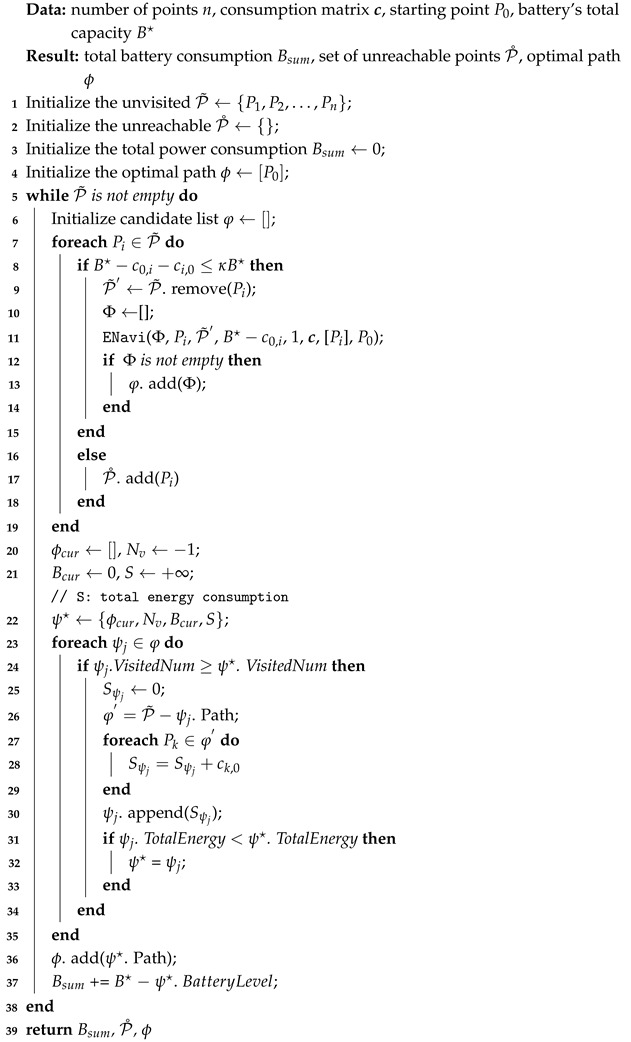



**Algorithm 2:** ENavi

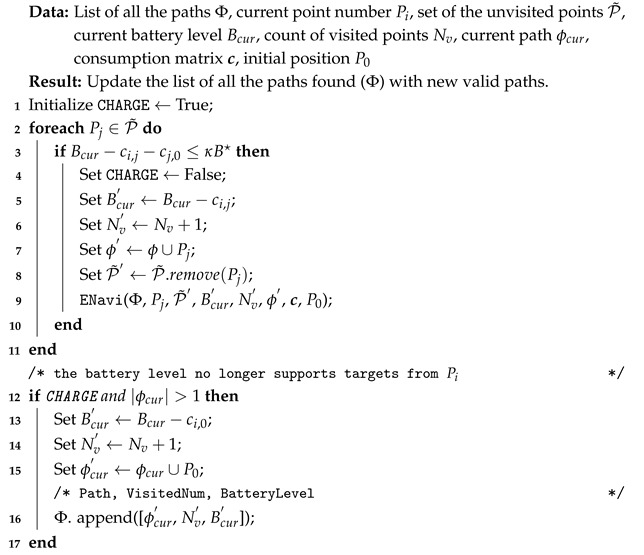



    It employs a depth-first search algorithm to ensure that the UAV visits as many edge nodes as possible within the energy constraints. The algorithm is divided into the following stages:

**(1) Initialization and verification:** While the UAV is stationed at a node, the navigation system checks neighboring nodes that have not been visited yet. For each node, it calculates whether the energy required to reach that node is sufficient and verifies if the UAV can return to the starting point after reaching it.

**(2) Recursive exploration:** When the energy conditions are met, the navigation system marks the current node as visited and generates a new list of unvisited nodes by removing the just-visited node. Then, the system sets the new node as the current node and recursively calls itself to continue searching for more accessible nodes. This process is akin to the UAV continuously flying forward, always ensuring a safe return.

**(3) Termination criteria:** The system stops running if the UAV cannot visit any more nodes (because of all the nodes being visited or insufficient energy), or the UAV has moved away from the starting point and cannot continue exploration, indicating that a valid path has been found.

**(4) Path recording:** Upon the completion of path exploration and if the UAV has visited at least one other node, ENavi records the path, the number of visited nodes, and the remaining energy, storing them in a global result list.

**(5) Backtracking mechanism:** After completing the current path exploration, the system returns to previous decision points to try other paths. Through this backtracking approach, the system can thoroughly examine all the possible paths and, ultimately, find the optimal solution, which is the path allowing the UAV to visit the maximum number of nodes.

**(6) Ensuring a safe return:** At each step, the system evaluates whether the UAV’s current energy is sufficient to meet the requirements for returning to the starting point, ensuring that no exploration depletes the energy entirely.

### 4.2. Complexity Analysis

The Hyperion algorithm employs a depth-first search with greedy expansion, dynamically evaluating the feasibility of future paths at each step. In the worst-case scenario, it explores all the permutations of the remaining points, resulting in the O(N!) time complexity, where *N* is the number of points, excluding the origin. But in practice, the battery constraints prune the search space significantly.

In Section 4.1, we can find that the ENavi algorithm serves as the core algorithm for UAV path planning in the outermost loop of the algorithm, assuming that the current number of remaining points is *k* (which is initially *N* and decreases incrementally). For each remaining point (*p*), the algorithm recursively calls the ENavi algorithm. In the worst-case scenario, the time complexity of this recursive process is O((k−1))! because all the permutations of the paths involving the remaining (k−1) points need to be explored. Therefore, the complexity of a single outer loop is k∗(k−1)!=k!. As k decreases from *N* to 1, the total time complexity can be expressed as $\sum_{k=1}^{N}k!$.

By demonstrating that $N!\leq \sum_{k=1}^{N}k!\leq 2N!$, the time complexity can be determined as O(N!). Initially, since $\sum_{k=1}^{N}k!=1!+\ldots +N!$, it is clear that $N!\leq \sum_{k=1}^{N}k!$. However, a detailed proof of the upper bound on the right-hand side will be provided as follows: When the number of remaining points is N=1, $\sum_{k=1}^{1}k!=1!=1\leq 2\cdot 1!=2$. Next, we use mathematical induction to prove this. Assuming that the upper bound holds when N=n, we now only need to prove that the formula still holds when N=n+1. When the number of remaining points is N=n+1,

(8) $$\sum_{k=1}^{n+1}k!\leq 2\left(n+1\right)!=n!\cdot 2\left(n+1\right)$$

According to the inductive assumption,

(9) $$\sum_{k=1}^{n+1}k!=\sum_{k=1}^{n}k!+\left(n+1\right)!\leq 2\cdot n!+\left(n+1\right)!=n!\left(n+3\right)$$

Combining formulas (8) and (9), we obtain

(10) n!(n+3)≤n!·2(n+1)

The simplification of formula (10) demonstrates that the inequality holds for N=n+1 under the condition 1≤n. Therefore, the original problem is proventhe and the time complexity can be determined as

(11) $$\sum_{k=1}^{N}k!\leq 2\cdot N!=O\left(N!\right)$$

## 5. Experiments

### 5.1. Preliminary

**Figure 1:**
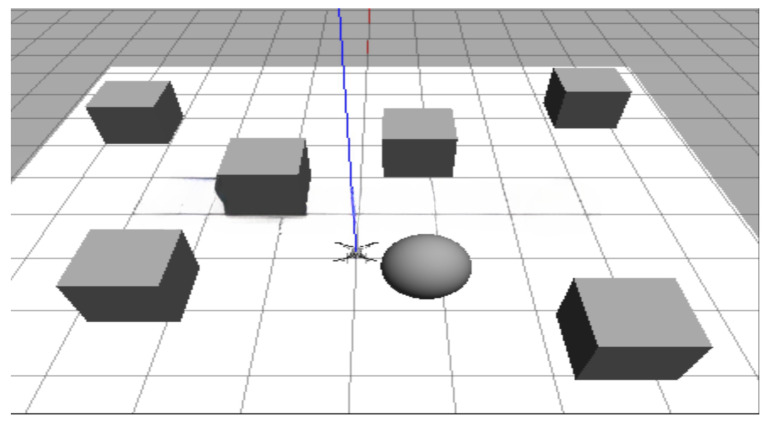
An illustration of the simulation environment.The blue line represents the possible flight path of the UAV.

In this section, we established a simulated experimental environment to evaluate the proposed method, as demonstrated in Figure 1, with Prometheus, using a three-dimensional spatial coordinate modeling approach to depict the positions of the end devices or edge nodes. Initially, a charging point, serving as the starting point for UAV flights, is manually selected. Subsequently, based on the location of the charging point, multiple edge nodes are randomly generated within a specified range. The information of these generated edge nodes includes their coordinate positions (x,y,z) and the amount of data each node needs to collect. Each node is then assigned a unique identifier, and a suitable flight path between every pair of nodes is planned according to the type of UAV and other configuration parameters. The energy consumption for flying between nodes is calculated to generate a flight energy consumption matrix.

The UAV takes off with a full battery from the starting point. Initially, the Hyperion algorithm is applied to determine the maximum number of nodes accessible to the UAV in the current state and to plan the path for node access. Subsequently, the UAV receives flight instructions and follows the planned path to collect data from the visited edge nodes. During this process, the energy consumption and the positions of the visited nodes are recorded. By conducting experiments in this simulated environment, a clearer analysis of the effectiveness and efficiency of the proposed method can be carried out, aiding in a deeper exploration of the prospects of UAV applications in edge data collection scenarios. In the analysis of the method’s effectiveness, a series of experiments was conducted by adjusting various environmental parameters to better understand their impacts. The important parameters used in the numerical experiments are presented in Table 1, were the parameter scope∼U[scope_min, scope_max] represents the range constraint coefficient for random node positions and is used to limit the energy consumption (*c*) associated with the flight distance. The energy consumption for all the nodes to reach the charging point must not fall below $scope\times B^{*}$. If scope>0.5, the position is identified as unreachable.

### 5.2. Introduction to Baselines

Previous research has primarily focused on simple, short-distance UAV-trajectory-planning scenarios [24,25,26]. However, in real-world settings, edge nodes that require UAVs for data collection are often located at considerable distances. In such long-distance scenarios, the limited battery capacity of UAVs necessitates multiple returns for recharging. Yet, most current studies have not effectively addressed path planning for this particular scenario. The classic Traveling Salesman Problem (TSP) requires visiting all the nodes in a single trip and returning to the starting point. In contrast, because of battery capacity constraints, our model requires multiple returns to the starting point (i.e., the charging station) for energy replenishment. As the Hyperion algorithm represents the first attempt at UAV-trajectory-planning scenarios with charging constraints, existing algorithms are not directly applicable because they were not designed for this specific application. Therefore, we select some similar, but representative, methods as baselines to compare with our proposed Hyperion method:

**(1) Global Optimal Strategy** (BEST): This method involves exploring all the possible paths to select the shortest path with the least overall energy consumption.

**(2) Hyperion-based Simplified Strategy** (Hyperion@1): A simplified version based on our strategy, Hyperion@1 considers the nearest node to the current location as the next node, unlike the full Hyperion method, which evaluates the overall factors.

(3) Modified Iterative-Deepening A* Method (MIDA*): This is an algorithm that enhances efficiency by incorporating upper-bound mechanisms and admissible lower-bound heuristics into an iterative deepening A* tree search [27].

**(4) Guided Genetic Algorithm** (GGA): It is a hybrid metaheuristic algorithm that combines genetic algorithms with a guided local search to optimize UAV flight paths and minimize battery and fuel consumption levels [28].

**(5) AC-based Strategy** (AC-ATP): It improves traditional ant colony (AC) algorithms by incorporating a pseudo-random proportional rule to enhance convergence [29].

**(6) DP-based Strategy** (DP-ATP): It decomposes complex problems into simpler subproblems for efficient resolution, saving processing time by storing subproblem solutions to avoid redundant computations [29].

### 5.3. Comparisons and Analysis

#### 5.3.1. Trajectory

**Figure 2:**
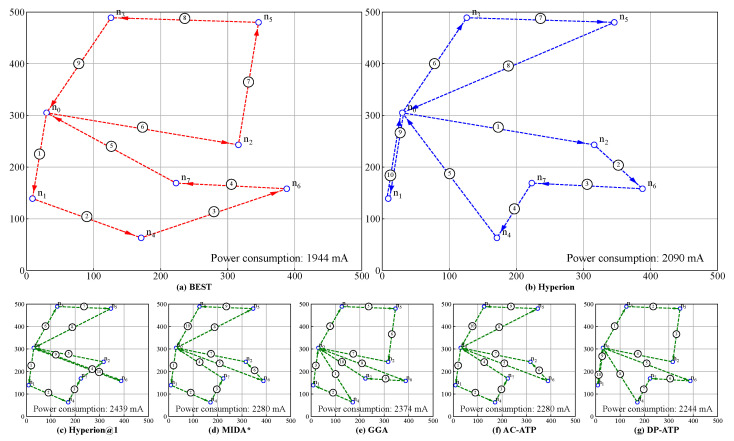
The trajectory paths of the Hyperion and baseline algorithms for the same parameters. The coordinates are not measured in meters (m) but are instead based on energy consumption, with the unit being milliampere-hours (mAh).The dotted lines in the figure represent the trajectory paths generated by the algorithm, and the numbers indicate the nodes.

From Figure 2, it is evident that the BEST method, as it traverses all the paths, always finds the optimal solution, resulting in the least energy consumption for the flight. However, because of the exhaustive search of all the possible paths, the computational time of the BEST method grows exponentially with an increase in the number of nodes, limiting its applicability.

On the other hand, although the Hyperion algorithm does not always generate the optimal path in the current scenario, it generally achieves results the closest to the BEST compared to other algorithms. In the scenario illustrated in Figure 2, the paths [$P_{0}$, $P_{4}$, $P_{7}$, $P_{6}$, $P_{2}$, $P_{0}$] and [$P_{0}$, $P_{1}$, $P_{4}$, $P_{6}$, $P_{7}$, $P_{0}$] have the same length, but the sum of the distances from nodes $P_{1}$, $P_{3}$, and $P_{5}$ to charging point $P_{0}$ is less than the sum of the distances from nodes $P_{2}$, $P_{3}$, and $P_{5}$ to charging point $P_{0}$. Therefore, the Hyperion algorithm selects the former path.

Hyperion@1, a simplified variant of the Hyperion algorithm, selects only the nearest node as the next step in the path. Consequently, the experimental results demonstrate that this method often converges to locally optimal solutions. In some cases, its excessive focus on immediate optimization leads to a significant increase in the overall energy consumption. For instance, in the scenario depicted in the figure, the energy consumption of Hyperion@1 is approximately 16.7% higher than that of Hyperion.

Comparing Figure 2b,d–f, we observe that MIDA*, GGA, and AC-ATP have performances similar to ours. The energy consumption of MIDA* is approximately 13.6% higher than that of Hyperion, the energy consumption of GGA is approximately 16.7% higher than that of Hyperion, and the energy consumption of AC-ATP is 9.1% higher than that of Hyperion. This is because of these algorithms being heuristic, where their effectiveness heavily relies on the heuristic rules used [30]. However, because the scenario proposed in this paper is not a common generic problem, specific rules tailored for this scenario have not been established yet, resulting in average performance.

Additionally, we can see that some paths generated by the dynamic-programming (DP) algorithm are the same as those generated by the Best method, but the remaining paths are entirely different. This discrepancy may arise from DP not being proficient in planning paths with energy constraints, leading to 15.4% higher energy consumption compared to the Best method and 14.4% higher than our Hyperion algorithm.

#### 5.3.2. Battery

**Figure 3:**
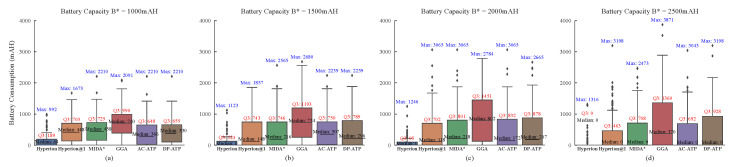
The energy consumption gaps among various algorithms with only the battery’s capacity being varied: (**a**) The battery capacity $B^{*}$ = 1000 mAh; (**b**) The battery capacity $B^{*}$ = 1500 mAh; (**c**) The battery capacity $B^{*}$ = 2000 mAh; (**d**) The battery capacity $B^{*}$ = 2500 mAh.

Figure 3 illustrates the distribution of the energy consumption differences between several approaches and the BEST algorithm across 100 random experiments, conducted under fixed spatial-scope and edge-node-clustering conditions while varying the battery’s capacity. As shown in the figure, Hyperion consistently maintains the narrowest gap with the BEST algorithm. Moreover, as the battery’s capacity increases, the gap between Hyperion and BEST narrows. This is because a higher battery capacity allows the Hyperion algorithm to consider more nodes during each path-planning iteration of the UAV. Consequently, for a constant number of nodes, the gap with BEST narrows. Notably, when the battery’s capacity reaches 2500 mAh, Hyperion achieves an energy consumption identical to that of BEST in 75% of the cases.

In contrast, the energy consumption gap between the Hyperion algorithm and the BEST algorithm remains relatively wide. Although this gap does not change significantly with increasing battery capacity, it narrows slightly when the battery’s capacity reaches 2500 mAh, with Hyperion matching BEST in 50% of the cases. However, because of its focus on immediate optimization, the variance of its distribution remains high.

Interestingly, the energy consumption gap between the GGA algorithm and the BEST algorithm initially widens with in increasing battery capacity but narrows slightly when the capacity reaches 2500 mAh. Despite this, GGA still underperforms compared to the other algorithms. This suboptimal performance may be attributed to the high computational cost and complex parameter selection of GGA, as this study did not conduct extensive parameter tuning for this algorithm. Additionally, the energy consumption gaps of the MIDA*, AC-ATP, and DP-ATP algorithms show only marginal improvements with increasing battery capacity. However, when the capacity reaches a threshold of 2500 mAh, a significant improvement occurs, with these algorithms matching the BEST algorithm in 50% of the cases.

Furthermore, the superiority of the Hyperion algorithm is also evident in the variance of its distribution. As the battery’s capacity increases, the variance of the energy consumption gap between Hyperion and BEST remains minimal. In contrast, the variance of the other algorithms increases significantly with higher battery capacities, indicating that they tend to produce suboptimal paths, often resulting in unnecessary detours.

**Figure 4:**
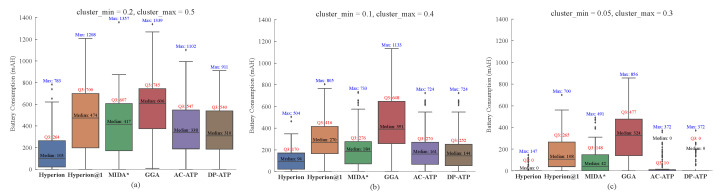
The performance of other algorithms compared to the BEST algorithm at different clustering degrees of edge nodes: (**a**) cluster_min = 0.2, cluster_max = 0.5; (**b**) cluster_min = 0.1, cluster_max = 0.4; (**c**) cluster_min = 0.05, cluster_max = 0.3.

Figure 4 presents the distribution of the energy consumption differences between BEST and the rest across 100 random experiments conducted at a fixed spatial scope and battery capacity while varying the clustering degree of the edge nodes, where **Median** represents the median value, **Q3** denotes the upper quartile (75th percentile) of the data range, and **Max** indicates the maximum value. From Figure 4a, it is evident that the Hyperion algorithm demonstrates a significant advantage over the other algorithms. Comparing the median gaps between these algorithms and BEST, Hyperion@1 exhibits a gap 4.6 times wider than Hyperion; MIDA*, 4.0 times wider; GGA, 5.9 times wider; ACO-ATP, 3.3 times wider; and ACO-DP, 3.0 times wider.

Furthermore, by comparing Figure 4a–c, it is observed that as the clustering degree of the nodes increases from low to moderate, the energy consumption gaps between all the other algorithms and the BEST algorithm narrow significantly. This is because higher clustering reduces the energy consumption between the nodes, allowing the algorithm to consider more nodes during each path-planning iteration for the drone. Consequently, for a constant number of nodes, the gap between the other algorithm and the BEST algorithm narrows. However, as the clustering degree further increases from moderate to high, the performances of the Hyperion and DP-ATP algorithms improve notably, with 75% of the random results matching those of the BEST algorithm. ACO-ATP performs slightly worse, while the results for Hyperion@1, MIDA*, and GGA show less pronounced changes. This indicates that these algorithms struggle to achieve the optimal performance under such conditions.

Additionally, Figure 4c reveals that when the clustering degree of the nodes reaches a sufficiently high level, Hyperion, ACO-ATP, and ACO-DP achieve energy consumptions identical to those of BEST in 75% of the cases. Even in this scenario, our Hyperion algorithm maintains a distinct advantage, as its distribution variance remains consistently low across multiple random trials, demonstrating its robustness and reliability.

#### 5.3.3. Completion Rate

**Figure 5:**
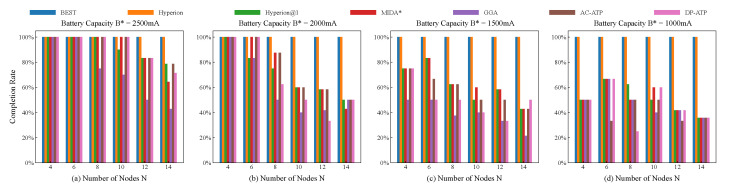
Comparison of the completion rates of the different approaches.

Figure 5 illustrates the impacts of various battery capacities and end device numbers (or node numbers) on the completion rates of UAVs within the same spatial range. As shown in Figure 5a, when the battery capacity is sufficiently high and the number of end devices is relatively low, all the algorithms achieve a 100% completion rate. However, as this number increases, only the BEST and Hyperion algorithms retain 100% completion rates. A comparison of the four subfigures in Figure 5 reveals that as the battery capacity decreases (from 2500 mA to 1000 mA), the completion rates of all the algorithms, except for BEST and Hyperion, exhibit a declining trend. This suggests that higher battery capacities enable the UAV to operate for longer durations, thereby increasing the likelihood of the task’s completion. Furthermore, as observed in Figure 5d, when the battery’s capacity is reduced to a certain threshold, the overall completion rates of Hyperion@1, MIDA*, GGA, AC-ATP, and DP-ATP tend to decrease as the number of nodes increases. However, because of the limited battery capacity, an anomalous phenomenon occurs, where the completion rate occasionally increases with a higher node number.

#### 5.3.4. Time Cost

**Figure 6:**
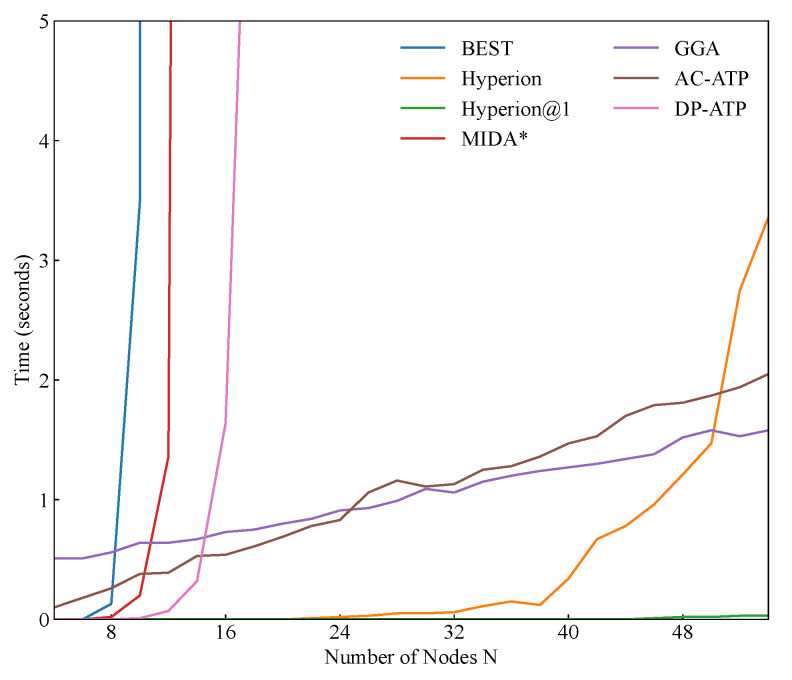
The time costs for different end-device numbers.

From Figure 6, we can observe the impact of varying the number of end devices on the execution times of the algorithms while keeping the other parameters fixed. It is evident that the BEST algorithm, which requires traversing all the possible paths, exhibits a rapid increase in the execution time. When the number of nodes exceeds 10, the execution time surpasses 100 s. Similarly, as the number of nodes increases, the search space of the MIDA* algorithm expands significantly, leading to a sharp rise in its execution time. Additionally, the DP-ATP algorithm shows a substantial increase in the execution time when the number of nodes exceeds 16. The proposed Hyperion algorithm maintains an execution time of 0.1 s when the number of nodes is below 40. However, beyond this threshold, the execution time increases rapidly. This is because within a fixed spatial range, as the number of nodes grows, the diversity will increase, and the probability of having a “neighbor” increases sharply. Consequently, Hyperion has more devices to consider during path planning, which prolongs the recursive computation time of the algorithm. Notably, when the number of devices exceeds 50, the execution time of the Hyperion algorithm exhibits an exponential growth trend. In contrast, Hyperion@1, which only needs to identify the nearest end device at each step, maintains a very short execution time. Even when the number of devices increases to 50, the execution time remains below 0.1 s. Furthermore, the execution times of the GGA and AC-ATP algorithms increase gradually as the number of nodes grows, indicating that these algorithms are less sensitive to changes in the number of devices.

**Figure 7:**
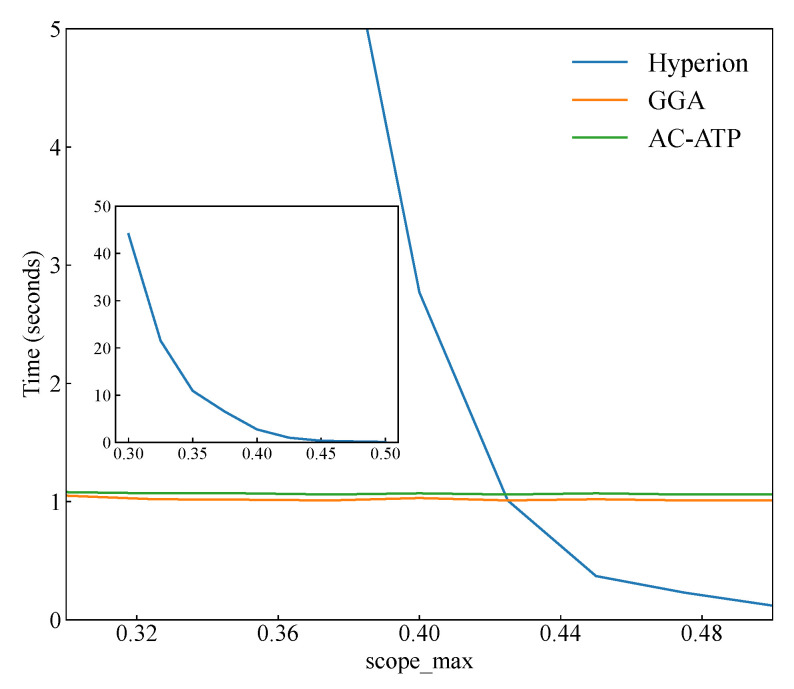
The time costs of different spatial ranges.

Additionally, the impacts of spatial range variations on the execution time become more pronounced only when the number of devices is sufficiently large. In Figure 7, we compare the performances of Hyperion, GGA, and AC-ATP possessing 30 end devices. It is evident that changes in the spatial range have almost no effect on the GGA and AC-ATP algorithms. This is because for these approaches, altering the positions of the nodes merely changes their numerical values, which does not affect their computational complexities. However, our proposed method, Hyperion, behaves differently. When the number of nodes remains constant, a narrower spatial range implies a denser distribution of devices in that space. This leads to a greater number of potential paths and more nodes along each path, both of which contribute to an increase in the algorithm’s execution time.

## 6. Learning-Based Planning Model

Given the escalating complexity as the scale of the problem increases, it is imperative to devise time-efficient solutions for creating alternative yet acceptable trajectories for the UAVs. This is where learning-based methodologies become essential.

In past research, many researchers would choose reinforcement learning (RL) to acquire the policy (π) by enabling an agent to interact with nodes and accumulate knowledge through numerous trials [31].

The limitations of RL-based approaches are also evident. A considerable number of training instances are often required for these approaches to ensure the model’s effectiveness, a requirement that poses significant challenges for certain constraint optimization problems (COPs) [32]. Current RL-based approaches predominantly focus on designing suitable reward functions tailored to specific problems, indicating limited generalizability [33]. The design of reward functions is particularly challenging in the context of large-scale instances that involve intricate constraint issues. This leads to substantial computational costs for training [34].

Essentially, the policy determines the subsequent optimal position. If a scoring model exists that can assign scores to all the potential next positions, we can consistently choose the destination with the highest score based on this metric. Consequently, we can represent the policy (π) as a model ($\pi (s,f_{i};\theta )$) with parameter θ that utilizes s, the UAV’s state, and $f_{i}$, the feature of any potential end device (*i*), to produce a score between 0 and 1, indicating the suitability of selecting $P_{i}$ as the next position. It is evident that this is a supervised learning task.

During the training of the supervised learning model, we generate decision data by running the Hyperion algorithm. As demonstrated by the experimental results in Section 5 (Figure 3, Figure 5 and Figure 6), Hyperion achieves outstanding performances in energy consumption and the task completion rate, closely approximating those of the BEST algorithm. Notably, in scenarios with sufficient battery capacity or highly clustered node distributions, Hyperion exhibits negligible energy consumption gaps compared to BEST in 75% of the experiments. Moreover, although the execution time of the BEST algorithm grows exponentially with increasing number of nodes (exceeding 100 s for just 10 nodes, making it impractical to generate an effective dataset), Hyperion incurs significantly lower time costs. On the basis of these validations, we choose Hyperion’s outputs as reliable labels for supervised learning. Thus, the initial step involves gathering relevant inputs and labels. This can be achieved in the following steps: Create a problem instance, where the environments of the end devices, the UAV, and the charging station are initialized. For each problem instance, execute the proposed method and document the specifics. For instance, if the UAV is currently positioned at $P_{k}$ in a problem instance and Hyperion suggests moving to $P_{j}$, then have π(s, $f_{j}$; θ) = 1 and π(s, $f_{i}$; θ) = 0 for all $P_{i}\in \tilde{P}-P_{j}$ as the ground truth or labels. By processing numerous problem instances, a dataset of inputs and labels can be collected for training. When a model is trained, we can use this model to make decisions: At a specific time (*t*), if the UAV is at point *k*, invoke $\pi_{\theta }$ for all the unvisited nodes $P_{j}\in \tilde{P}$. Obtain scores ranging from 0 to 1 for all $P_{j}\in \tilde{P}$, and select the one with the highest score as the subsequent target.

**Figure 8:**
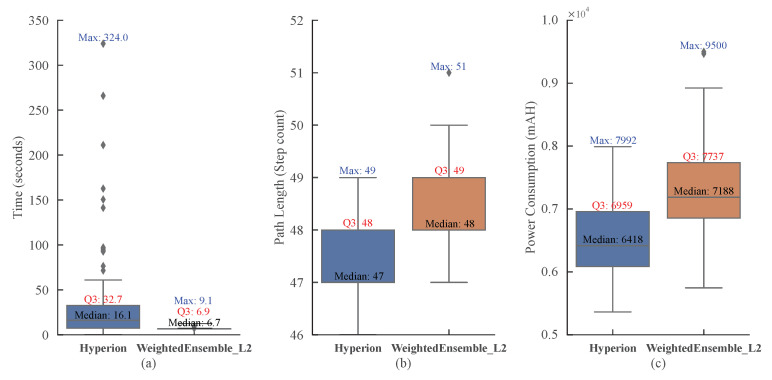
Comparison of Hyperion and Learning-Based Methods across different aspects: (**a**) Execution time; (**b**) Path lengths; (**c**) Energy consumption.

In this work, we construct a weighted-ensemble model by combining multiple base models through weighted aggregation to accomplish the task [35]. First, multiple base models of different types (e.g., random forests, gradient-boosting trees, and neural networks) are trained. Next, the performances of these models are evaluated in a validation set, and a weight is assigned to each model, typically optimized based on the model’s performance. Finally, the predictions of these models are integrated using weighted averaging (for regression tasks) or weighted voting (for classification tasks) to produce a more accurate and robust final prediction. Weighted ensemble is a data-driven approach trained on historical trajectories generated by Hyperion. It predicts the next optimal node based on features such as energy consumption and spatial relationships. Figure 8 presents the results of comparative experiments between the proposed Hyperion and the trained model weighted ensemble within 100 runs (the weighted ensemble is composed of a LightGBM and an MLP with weights of 0.4 and 0.6), conducted at a relatively large problem scale with a device number of N = 40 and a spatial range of scope_max = 0.4. As clearly shown in Figure 8a, the execution time of the weighted ensemble for path prediction is highly stable, ranging between 6 and 9 s. In contrast, the execution time of Hyperion is influenced by multiple parameters, with higher values exceeding 300 s and lower values of around 16 s. Figure 8b compares the path lengths, revealing that when N = 40, the weighted ensemble typically requires one additional step compared to Hyperion, indicating a relatively minor difference. Figure 8c illustrates the energy consumption comparison, where the median values from multiple experiments show that the weighted ensemble consumes 11.9% more energy than Hyperion. Therefore, when considering the execution time, path length, and energy consumption collectively, employing a learning-based model for path prediction emerges as a feasible alternative.

In low-density end-device scenarios, the Hyperion algorithm achieves energy efficiency close to the theoretical optimum. This characteristic makes it an ideal choice for small-to-medium-scale IoT applications. However, as the number of end devices increases, Hyperion’s time complexity exhibits exponential growth, leading to significant latency penalties. In large-scale scenarios, the weighted-ensemble algorithm substantially reduces the time complexity by sacrificing marginal energy efficiency in exchange for significantly reduced execution time. This characteristic grants the weighted ensemble stronger practicality in time-sensitive or large-scale problem scenarios.

## 7. Conclusions and Future Work

In this paper, we addressed the critical challenge of efficiently collecting data from edge nodes in extreme scenarios, where traditional wireless networks are unreliable or unavailable, which leads to scenarios where UAVs are used to collect distributed data on end devices. We proposed a novel UAV-trajectory-planning algorithm, Hyperion, which incorporates energy constraints and a greedy strategy to optimize flight paths, ensuring that the UAV can complete data collection tasks while maintaining *sufficient battery power* for a *safe return*. Our approach not only enhances the reliability and safety of UAV operations but also improves energy efficiency and adaptability in complex environments. Through extensive simulations, we demonstrated that Hyperion outperforms several baseline algorithms in terms of energy consumption, completion rate, and execution time. Specifically, Hyperion consistently achieves results the closest to the global optimal strategy (BEST) while significantly reducing computational complexity. Additionally, we introduced a learning-based planning model, weighted ensemble, which offers a time-efficient alternative for large-scale problems, albeit with a slight tradeoff in energy efficiency.

Although our proposed methods show promising results, several avenues for future research remain, paving the way for broader adoption in various applications, including disaster responses, remote monitoring, and autonomous systems:

**(1) Dynamic Environments**: The current approach assumes a static environment. Future work could explore dynamic scenarios where edge nodes or obstacles move, requiring real-time adaptation of UAV trajectories [36].

**(2) Multi-UAV Coordination**: Extending the framework to multiple UAVs could further enhance data collection efficiency [37]. Coordinating multiple UAVs to avoid collisions and optimize collective energy consumption presents a challenging yet valuable direction.

**(3) Real-World Deployment**: Validating the proposed algorithms in real-world scenarios with physical UAVs and edge devices would provide practical insights and highlight potential areas for improvement.

## Figures and Tables

**Table 1:** The settings of the experiments.

Item	Value	Item	Value
*B*	1000.0	*N*	8
scope_min	0.1	scope_max	0.5
cluster_min	0.1	cluster_max	0.5

## Data Availability

The raw data supporting the conclusions of this article will be made available by the authors on request.
